# Early tracheostomy in closed head injuries: experience at a tertiary center in a developing country – a prospective study

**DOI:** 10.1186/1471-227X-5-8

**Published:** 2005-10-14

**Authors:** Chintamani, Jotinder Khanna, JP Singh, Pranjal Kulshreshtha, Pawan Kalra, Binita Priyambada, RS Mohil, Dinesh Bhatnagar

**Affiliations:** 1 Department of surgery, Vardhman Mahavir Medical College, Safdarjang Hospital, New Delhi-110023, India

## Abstract

**Background:**

An important factor contributing to the high mortality in patients with severe head trauma is cerebral hypoxia. The mechanical ventilation helps both by reduction in the intracranial pressure and hypoxia. Ventilatory support is also required in these patients because of patient's inability to protect the airway, persistence of excessive secretions, and inadequacy of spontaneous ventilation. Prolonged endotracheal intubation is however associated with trauma to the larynx, trachea, and patient discomfort in addition to requirement of sedatives. Tracheostomy has been found to play an integral role in the airway management of such patients, but its timing remains subject to considerable practice variation. In a developing country like India where the intensive care facilities are scarce and rarely available, these critical patients have to be managed in high dependency cubicles in the ward, often with inadequately trained nursing staff and equipment to monitor them. An early tracheostomy in the selected group of patients based on Glasgow Coma Score(GCS) may prove to be life saving.Against this background a prospective study was contemplated to assess the role of early tracheostomy in patients with isolated closed head injury.

**Methods:**

The series consisted of a cohort of 50 patients admitted to the surgical emergency with isolated closed head injury, that were not considered for surgery by the neuro-surgeon or shifted to ICU, but had GCS score of less than 8 and SAPS II score of more than 50. First 50 case records from January 2001 that fulfilled the criteria constituted the control group. The patients were managed as per ATLS protocol and intubated if required at any time before decision to perform tracheostomy was taken. These patients were serially assessed for GCS (worst score of the day as calculated by senior surgical resident) and SAPS scores till day 15 to chart any changes in their status of head injuries and predictive mortality. Those patients who continued to have a GCS score of <8 and SAPS score of >50 for more than 24 hours (to rule out concussion or recovery) underwent tracheostomy.

All these patients were finally assessed for mortality rate and hospital stay, the statistical analysis was carried out using SPSS10 version.

The final outcome (in terms of mortality) was analyzed utilizing chi-square test and p value <0.05 was considered significant.

**Results:**

At admission both tracheostomy and non-tracheostomy groups were matched with respect to GCS score and SAPS score.

The average day of tracheostomy was 2.18 ± 1.0038 days.

The GCS scores on days 1, 2, 3, 4, 5, 10 between tracheostomy and non-tracheostomized group were comparable. However the difference in the GCS scores was statistically significant on day 15 being higher in the tracheostomy group.Thus early tracheostomy was observed to improve the mortality rate significantly in patients with isolated closed head injury

**Conclusion:**

It may be concluded that early tracheostomy is beneficial in patients with isolated closed head injury which is severe enough to affect systemic physiological parameters, in terms of decreased mortality and intubation associated complications in centers where ICU care is not readily available. Also, in a selected group of patients, early tracheostomy may do away with the need for prolonged mechanical ventilation.

## Background

An important factor responsible for the high mortality in patients with severe head trauma is cerebral hypoxia. Mechanical ventilation is often required because of patient's inability to protect the airway, persistence of excessive secretions, and inadequacy of spontaneous ventilation [1]. Tracheostomy plays an integral role in the airway management of such patients, but its timing remains subject to considerable practice variation [1-4]. The complications associated with prolonged endotracheal intubation are increasingly being recognized and include injury to the larynx, trachea, and patient discomfort [5-13,16-18]. In addition, endotracheal intubation often requires the administration of systemic sedation, with attendant complications. Finally the incidence of ventilator-associated pneumonia is related directly to the duration of mechanical ventilation – a complication that carries significant morbidity and mortality [19-22].

Paradoxically, although tracheostomy is frequently recommended in closed head injury patients, few studies have been carried out to assess the importance in this group of patients. Many studies recommend early tracheostomy to avoid serious oropharyngeal and laryngeal injury occurring from prolonged translaryngeal intubation though limited data is available to define the impact of early tracheostomy on duration of mechanical ventilation and hospital stay [1,4,5,9].

In a developing country like India, where even a tertiary care center like ours, is short of appropriate ICU facilities for patients with severe closed head injuries for all patients, these critical patients have to be managed in high dependency cubicles in the ward, often with inadequately trained nursing staff and equipment to monitor them.

We had observed that patients with severe head injury very frequently required prolonged intubation, primarily for airway protection after initial few days and due to fluctuating changes in airway reflexes patients often struggled with endotracheal tubes and required frequent use of sedatives.

With the underlying belief that early tracheostomy is beneficial, we searched the literature to find those objective factors (GCS and SAPS) [1,3] which can be used early to separate those patients who will ultimately require tracheostomy from those that will not.

## Methods

### Settings

The study was performed at a major tertiary care trauma centre in New Delhi, India. This 2200-bed hospital has an 8-bed medical/surgical ICU staffed by full-time, on-site intensivists 24 hours a day and 7 days a week. The hospital has a designated trauma service, including a consultant surgeon, available 24 hours a day. Medical care in the ICU is provided by the ICU team, with the trauma team being responsible for surgical aspects of care. In addition there is a 4-beded neuro-ICU for post-operative neurosurgical patients staffed by neurosurgeons. The surgical ward has 37 beds with a 6-bed high dependency cubicle.

The study was carried out following review with Institutional Review Board from July 2003 to November 2004. The series consisted of consecutive 50 patients who fulfilled the criteria of the study and underwent tracheostomy. First 50 case records from January 2001 that fulfilled the criteria of the study constituted the control group.

This study consisted of cohort of all patients admitted to surgery emergency with isolated closed head injury, that were not considered for surgery by the neuro-surgeon or shifted to ICU, but had GCS score of less than 8 and SAPS II score of more than 50 (predictive mortality more than 50%, calculated at ). The patients were managed as per ATLS protocol and intubated if required at any time before decision to perform tracheostomy was taken. Patients underwent NECT scan to find out any intracranial bleed and were managed as per the advice of neurosurgeon.

Those patients who continued to have a GCS score of <8 and SAPS score of >50 for more than 24 hours (to rule out concussion or recovery) were to undergo a tracheostomy with a standardized technique by the surgical resident. The following patients were excluded from the study

1) Children less than 12 years of age

2) Patients who underwent neurosurgical intervention, as these patients were later shifted to neuro-ICU

3) Patients who were shifted to the ICU as ventilator and / or ICU bed were available

4) Patients with poly trauma with other injuries besides closed head injuries, so as to constitute a homogenous cohort.

The control group was selected from previous admission records and only those cases were included in whom complete data so as to calculate serial SAPS II score were available, besides fulfilling the above criteria. This was to achieve a score matched control group as comparable to study group as possible and in order to eliminate confounding factors.

All these patients were assessed for mortality rate and hospital stay and 30 days (from the time of admission) mortality was taken in to consideration. These patients were also serially assessed for GCS (worst score of the day as calculated by senior surgical resident) and SAPS scores till day 15 to chart any changes in their status of head injuries and predictive mortality.

Surgical resident in the ward performed tracheostomy by the standardized technique using a high volume, low pressure cuffed tracheostomy tube.The patient was shifted to high dependency cubicle for monitoring and 2–3 hourly suction of the tracheostomy by the staff nurse. The nurse-patient ratio in the high dependency cubicle is 1:6. If patient required ventilatory support a small KV ventilator was arranged from ICU to supply 40% oxygen at a fixed rate and airway pressure. If that was not available the patients were put on continued Ambu-bag ventilation till a ventilator was arranged. Other resuscitation was carried as per the instruction of the ICU resident and neurosurgical resident, including use of sedatives.

All patients were to undergo flexible laryngoscopy within 24 hours of tracheostomy by an ENT senior resident for evaluation of endotracheal injury and at the time of extubation or tracheostomy removal.

A central venous access was established and daily routine investigations were sent for these patients. As and when the patient stabilized (SAPS II score <50), patient was placed in the general ward with patient-nurse ratio of 1:15. A naso-gastric tube was placed and feeding started initially along with intravenous supplement and gradually only liquid diet through naso-gastric tube, providing 2200Calories a day was started. This was done till the patient gained adequate consciousness or oropharyngeal reflexes. The patient was also placed on water mattress with nursing attendants log rolling the patients every hour to prevent bedsores. The patients' attendants were gradually introduced to nursing care of the patient and taught precautions regarding hygiene and feeding. Staff nurses or surgical residents did tracheostomy care and dressing of the bedsores on a regular basis.

Special care was taken to prevent constipation and early switch from Foley's catheter to condom catheter drainage was done. Catheter care was done as long as Foley's catheter was in-situ.

Complications like fever, cough, blocked tracheostomy tube with sputum, bedsores and or any other source of sepsis were dealt with as per standard protocol of culture sensitivity and appropriate antibiotic cover.

The patients were discharged only after

1) They regained adequate consciousness (GCS >13);

2) Patients not only improving in the GCS score but had a good SAPS II score Meanwhile attendants were adequately trained in patient care and patient gradually weaned off tracheostomy.

Patients were divided into two groups: those that under went tracheostomy (T group) and those that did not (NT group). All the data was collected and comparisons between the two groups for continuous variables are expressed as means ± standard error of the mean, and were compared using two-tailed t-tests for unequal variance. Categorical variables are expressed as absolute and relative frequencies, and were compared using χ^2 ^tests. P ≤ 0.05 were considered statistically significant.

## Results

Out of the 9071 patients admitted with head injury during the period of our study, 542 had isolated closed head injuries, of which 56 patients satisfied the criteria of our study. 6 patients could not be included due to incomplete case records. The remaining 50 constituted the cases, i.e. those undergoing early tracheostomy (group T). Of the 1125 case records of patients admitted between January 2001–December 2003 which were studied, the first 50 satisfactory case records were taken as the control (group NT). In this study the distribution of patients in the two groups was matched with respect to confounding factors. The average age in the tracheostomy group (T) was 34.54 ± 12.7737 years and in the non-tracheostomy group (NT) was 32.98 ± 11.1435 years. Using two-tailed t-test for unequal variance p value was 0. 5209, which is not significant. Similarly sex distribution in the two groups was matched. The male: female ratio in the T group was 37:13 and in the NT group was 33:17. The two groups were analyzed using chi-square test. The cell chi value was 0.11, degree of freedom was 1 and the p value was 0.4571, which is not significant.

The comparison of GCS and SAPS was done for Days 1, 2, 3, 4, 5, 10 and 15 between tracheostomy and the non-tracheostomy group. The GCS score on day 1 of admission was 4.06 ± 1.0278 (T) and 4.14 ± 0.9382 (NT); comparing the two groups using two-tailed t-test for unequal variance, p value was 0.6882, which is not significant [<0.05 taken as significant]. Similarly SAPS score on day 1 of admission was 59.34 ± 2.7247 (T) and 58.94 ± 2.2127 (NT); comparing the two groups using two-tailed t-test for unequal variance, p value was 0.6017, which is not significant. Thus at admission both tracheostomy and non-tracheostomy groups were matched with respect to GCS score and SAPS score.

The average day of tracheostomy was 2.18 ± 1.0038 days.

**Figure 1:**
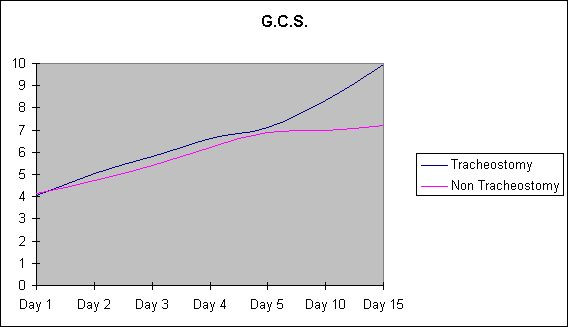
The Glasgow coma score of the two groups from day 1 to days 15.

**Figure 2:**
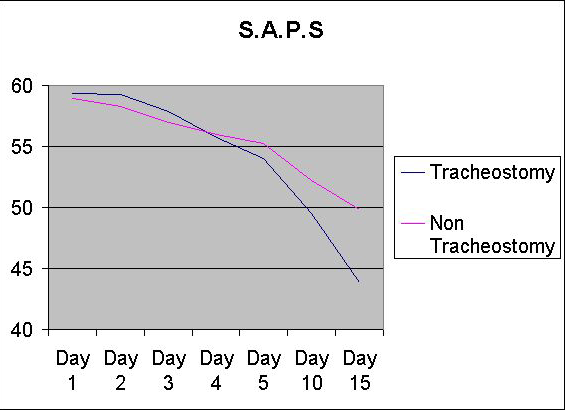
The SAPS score of the two groups (tracheostomized and non-tracheostomized) from day 1 to days 15.

The GCS scores on days 1, 2, 3, 4, 5, 10 between tracheostomy and non-tracheostomy group were comparable, the p value being not significant between the two groups. However GCS scores were statistically significant on day 15 being higher in the tracheostomy group indicating improvement with tracheostomy (Table 1, Figure 1). Similarly the SAPS score on days 1, 2, 3, 4, 5 were comparable between tracheostomy and non-tracheostomy groups, the p value being not significant between the two groups. However, SAPS score on days 10 and 15 were statistically significant, being lower in the tracheostomy group indicating improvement with tracheostomy (Table 2, Figure 2).

**Table 1:** Comparison of sequential G.C.S. between tracheostomised and non-tracheostomised groups.

	Tracheostomy	Non Tracheostomy	p Value
Day 1	4.06 ± 1.0278	4.14 ± 0.9382	0.6882
Day 2	5.04 ± 1.1825	4.74 ± 1.1800	0.2117
Day 3	5.82 ± 1.6815	5.40 ± 1.8617	0.2560
Day 4	6.62 ± 1.9596	6.21 ± 2.3211	0.3681
Day 5	7.12 ± 2.7472	6.91 ± 2.9671	0.7324
Day 10	8.35 ± 3.1983	7.00 ± 3.4935	0.0723
Day 15	9.97 ± 3.0477	7.20 ± 3.3506	0.0009

**Table 2:** Comparison of sequential S.A.P.S. between tracheostomised and non-tracheostomised groups.

	Tracheostomy	Non Tracheostomy	p Value
Day 1	59.34 ± 2.7247	58.94 ± 2.2127	0.6016
Day 2	59.24 ± 2.4294	58.30 ± 2.3515	0.7243
Day 3	57.88 ± 3.4850	56.97 ± 2.1617	0.1303
Day 4	55.70 ± 3.8587	55.97 ± 2.9302	0.6965
Day 5	54.04 ± 4.4856	55.25 ± 3.7422	0.1961
Day 10	49.48 ± 6.1954	52.20 ± 5.8929	0.0460
Day 15	43.86 ± 8.1665	49.83 ± 7.2391	0.0028

Besides the improvement in the GCS and SAPS scores the end point of the study was to see whether tracheostomy led to decrease in mortality rate when compared with similar controls and whether there was reduction in hospital stay in tracheostomised patients. The final outcome (in terms of mortality) was analyzed utilizing chi-square test. The cell chi was 1.30 and 1.141, the degree of freedom was 1 and p was less than 0.05, which is significant (Table 3). The mortality in the T group was 36% compared to that of 58% in the NT group. This also compares favorably with the predictive mortality of >50% with SAPS II. Thus doing early tracheostomy improved significantly the mortality rate in patients with isolated closed head injury.

**Table 3:** Chi square table for outcome in the tracheostomised and non-tracheostomised groups.

	Discharged	Expired
	32	18
Tracheostomy	26.5	23.5
	1.141	1.30
	21	29
Non Tracheostomy	26.5	23.5
	1.141	1.30

Degree of freedom = 1

Overall cell chi = 4.88

p = 0.0275 (significant)

Hospital stay was analyzed separately for the patients who were discharged or those who died during the course of stay in hospital. The average day of mortality was 19.4130 ± 7.0476 (T) and 18.55 ± 9.7469 (NT); comparing the two groups using two-tailed t-test for unequal variance, p value was 0.6244, which is not significant. Similarly average day of discharge was 19.78 ± 6.9261 (T) and 18.66 ± 9.7834 (NT); comparing the two groups using two tailed t-test for unequal variance, p value was 0.5157, which is not significant (table 4). Performing early tracheostomy did not change length of hospital stay significantly in either patients who were discharged or those who expired during hospital stay. Probably this was so because abundant caution was taken to see that the patient is weaned off the tracheostomy and the attendants are adequately trained to perform nursing care of these patients. The very limited rehabilitation facilities meant that the patients had to undergo rehabilitation while they were hospitalized, prolonging further the hospital LOS.

**Table 4:** Comparison of hospital stay in the tracheostomised and non-tracheostomised groups.

	Tracheostomy	Non Tracheostomy	p Value
Day of discharge	19.78 ± 6.9261	18.66 ± 9.7834	0.5157
Day of death	19.41 ± 7.0476	18.55 ± 9.7469	0.6244

## Discussion

A tracheostomy is a proven adjunct in the care of head injury patients [5,8,9,12]. Tracheostomy provides an early airway protection and seems to decrease the need for prolonged mechanical ventilatory support. Secondly, severe head injury patients require a prolonged time for recovery and the airway reflexes are rarely optimal [3]. The association between the duration of intubation and risks of laryngotracheal injury is another important consideration in the timing of tracheostomy.

The tracheostomy tube facilitates pulmonary toilet and oral hygiene and has been shown to reduce the incidence of ventilator-associated pneumonia [5]. Furthermore, a tracheostomy tube is less noxious for the patient emerging from coma and sedation can be more easily weaned off. In addition, tracheostomy reduces significantly the physiological dead space of ventilation and thereby the work of breathing. This is even more beneficial in a patient having labored breathing or deteriorating respiration and may prevent the use of mechanical ventilation altogether. Because of these benefits, early tracheostomies have been shown to reduce hospital stay. However to realize the benefits of early tracheostomy without performing unnecessary tracheostomies, appropriate patients must be identified early at the time of admission. Early oxygenation and ventilatory abnormalities can predict the need for tracheostomy [19]. However, head injury patients primarily require airway protection and not necessarily ventilatory support for pulmonary failure.

A study by Major et al [1] showed that using objective scores such as GCS (less than seven) and SAPS (more than fifteen) score could aid in identifying those patients who will eventually require a tracheostomy for prolonged airway protection after blunt head trauma with high positive predictive value. However, they used day 4 scores of GCS and SAPS to determine the need for tracheostomy and performed the tracheostomy only after day 5. This way they not only excluded patients who were extubated but even those who died early, thus removing an important cohort who could benefit from tracheostomy. This skewed mortality data as severe head injury is associated with early mortality. In addition, these patients were admitted in ICU and were intubated already as per ATLS standards. Positive predictive value for GCS and SAPS score was 71%, and negative predictive value was 83%. Gurkin et al [6] found two admission criteria (GCS <9 and ISS >24) to be predictive of the need for tracheostomy in those patients that remained intubated on day 7. Rodriguez et al [9] noted a significant decrease in ventilator days and ICU days and hospital length of stay in patients who underwent tracheostomy within five days of intubation.

In a study by Sugerman et al [4] several major trauma centers refused to participate in early tracheostomy (5 to 7 days) trial because they felt strongly, like we do, that either all severly injured patients should undergo tracheostomy within 2 to 3 days after injury or tracheostomy was not necessary for as long as 3 to 4 weeks after injury. In their study they found that head injury patients who underwent early tracheostomy had higher APACHE III scores but there was no difference in ICU LOS, pneumonia and death in these patients as compared to patients with late tracheostomy.

Lesnik *et al *[5] retrospectively reviewed 101 adult patients with blunt injuries, 32 had tracheostomy within first 4 days and 69 underwent tracheostomy after 4 days. The author found that mean duration of ventilatory support was 6.0 days in early tracheostomy group versus 20.6 days in the late tracheostomy group (p < 0.001).

In a study by Bouderka et al a prospective study was conducted in patients with admission GCS of 8 or less, cerebral contusion on CT scan and GCS score of less than 8 on day 5. These patients were randomized into early tracheostomy and prolonged intubation. Besides demographic data admission scores SAPS, ICU stay duration of mechanical stay was compared. They concluded that early tracheostomy decreased total days of mechanical ventilation (p = 0.02). They agreed that choosing their criteria had the limitation of high mortality during the first week of hospitalization. They suggested that most of the patients did not require mechanical ventilatory support but were intubated mainly for airway protection. Early tracheostomy may provide an early alternative for airway protection and assist in early termination of mechanical ventilatory support and there fore reduce hospital stay for these patients.

In our opinion using admission data to support a decision several days later is flawed. This diminishes the benefits of early tracheostomy and makes the decision more straightforward and increases the chances of laryngotracheal injuries due to intubation.

Early tracheostomy may assist in early termination of mechanical ventilation and therefore, reduce the hospital stay and mortality. Currently, the decision to proceed to early tracheostomy is based on the attending trauma surgeon's preference. The consensus conference of 1989 recommended conversion to tracheostomy if the anticipated need for mechanical ventilation is more than 21 days [15]. Such practice was based on earlier reports showing high tracheal stenosis rates with tracheostomy as compared with endotracheal intubation [13]. However, the incidence of tracheal stenosis has decreased substantially with recognition of its aetiology and improvements in tracheostomy materials, design and management, particularly with the use of high-volume, low-pressure cuffs. Also, the complications associated with prolonged endotracheal intubation are increasingly being recognized, including injury to the larynx and trachea [16-18], and patient discomfort. In addition, endotracheal intubation often requires the administration of systemic sedation, with attendant complications.

Despite evidence to support the utility of early tracheostomy, few recommendations exist to facilitate identification of appropriate patients.

In a study by Arabi et al [2], 136 patients underwent tracheostomies, of which only 29 were early (seven days). The duration of mechanical ventilation was significantly shorter with early tracheostomy (mean ± standard error: 9.6 ± 1.2 days versus 18.7 ± 1.3 days; *P *< 0.0001). Similarly, ICU LOS was significantly shorter (10.9 ± 1.2 days versus 21.0 ± 1.3 days; *P *< 0.0001). Following tracheostomy, patients were discharged from the ICU after comparable periods in both groups (4.9 ± 1.2 days versus 4.9 ± 1.1 days; not significant). ICU and hospital mortality rates were similar. Using multivariate analysis, late tracheostomy was an independent predictor of prolonged ICU stay (>14 days). The very low mortality seen in the patients we studied may be explained by selection of proper candidates for tracheostomy, excluding those patients who were unlikely to survive. Hospital LOS in these patients was prolonged, reflecting their severe injuries that required lengthy rehabilitation periods.

Strengths of our study include prospective data collection ensuring complete data. The cohort was homogenous in that the decision for tracheostomy was not affected by other injuries (maxillofacial, neck) which may mandate early tracheostomy. Similarly the outcome in either group remained uninfluenced by systemic injuries. Our study included all patients with severe head injury (including those with early mortality) by virtue of doing early tracheostomy, unlike other studies [1-4,10,11] which excluded such patients by doing tracheostomy on day 5 onwards. By taking SAPS II as a criterion, we tried not to overdo tracheostomies by including only those patients in whom the head injury was severe enough to affect systemic physiological parameters. Lastly, we have taken serial scores as criteria for tracheostomy rather than admission scores. However data extraction and analysis was retrospective. Because the database was not designed specifically to examine tracheostomy practices, certain issues were not documented, such as comparison with intubation and the type of tracheostomy done. Also, we did not compare the morbidity in the two groups. In addition, the study was conducted from one centre. A large multicentre randomized controlled trial in which patients are randomized to early versus late tracheostomy would be the ideal way to test the impact of procedure timing on resource utilization.

## Conclusion

We conclude that early tracheostomy is beneficial in patients with isolated closed head injury, severe enough to affect systemic physiological parameters, in terms of decreased mortality and intubation associated complications in centers where ICU care is not readily or easily available. Also, in a selected group of patients, early tracheostomy may actually do away with the need for prolonged mechanical ventilation.

## Competing interests

The author(s) declare that they have no competing interests.

## Authors' contributions

CM, the principal author designed the study and was the surgeon in charge monitoring the management of these patients, JK contributed to the designing of study and statistical analysis., PK, JP, BP, P kalra, were the residents in charge of the management of these cases.RSM, DB were the senior surgeons also involved in the management of the cases.

## Pre-publication history

The pre-publication history for this paper can be accessed here:


